# Intraepithelial lymphocytes in human oral diseases

**DOI:** 10.3389/fimmu.2025.1597088

**Published:** 2025-05-08

**Authors:** Dong-Yang Zhou, Chao-Fan Bao, Gang Zhou

**Affiliations:** ^1^ State Key Laboratory of Oral & Maxillofacial Reconstruction and Regeneration, Key Laboratory of Oral Biomedicine Ministry of Education, Hubei Key Laboratory of Stomatology, School & Hospital of Stomatology, Wuhan University, Wuhan, China; ^2^ Department of Oral Medicine, School and Hospital of Stomatology, Wuhan University, Wuhan, China

**Keywords:** intraepithelial lymphocytes, oral lichen planus, oral squamous carcinoma, periodontal disease, graft-versus-host disease, primary Sjogren’s syndrome

## Abstract

**Objective:**

As a distinctive subset of T cells, intraepithelial lymphocytes (IELs) are found in the epithelium of mucosal barrier and serve as the primary defenders of the intestinal mucosal immune system. IELs exhibit phenotypic and functional diversity with high expression of activated marker molecules, tissue-homing integrins, NK cell receptors, cytotoxic T cell-related molecules, and cytokines. Meanwhile, IELs demonstrate differentiation plasticity, antigen recognition diversity, self-reactivity, and rapid “memory” effect, which enable them to play a crucial role in regulating responses, maintaining mucosal barriers, promoting immune tolerance, and providing resistance to infections. In addition, IELs have been explored in autoimmune diseases, inflammatory diseases, and cancers. However, the specific involvement and underlying mechanisms of IELs in oral diseases have not been systematically discussed.

**Methods:**

A systematic literature review was conducted using the PubMed/MEDLINE databases to identify and analyze relevant literatures on the roles of IELs in oral diseases.

**Results:**

The literature review revealed the characteristics of IELs and emphasized the potential roles of IELs in the pathogenesis of oral lichen planus, oral cancers, periodontal diseases, graft-versus-host disease, and primary Sjogren’s syndrome.

**Conclusion:**

This review mainly focuses on the involvement of IELs in oral diseases, with a particular emphasis on the main functions and underlying mechanisms by which IELs influence the pathogenesis and progression of these conditions.

## Introduction

Being a distinctive subgroup of T cells located in the mucosal epithelium, intraepithelial lymphocytes (IELs) differ from other T cells in terms of tissue distribution, phenotype, subgroup differentiation, and function (1). As a class of killing lymphocyte population, IELs exhibit potent, rapidly activated cytolytic and immunomodulatory effectors (2). Meanwhile, IELs resist pathogenic infections by supporting epithelial cells and regulating the barrier function of acquired and innate immunity (3).

By far, emerging studies have explored the role of IELs in chronic inflammation, autoimmune diseases, and tumors as a potential regulator of pathogenesis and a future therapeutic target. Moreover, IELs’ involvement in oral diseases has gradually garnered public attention. In oral lichen planus (OLP), significant infiltration of CD8^+^ IELs were observed (4). IELs infiltration was found in oral squamous cell carcinoma and was strongly associated with prognosis (5–7). γδ IELs were the first line of defense against luminal microorganisms and could induce inflammatory factors, which mediated the development and progression of periodontitis (8). IELs were increased in the gallbladder of patients with hematopoietic stem cell transplantation (HCT) (9). Currently, a scoring system for oral graft-versus-host disease (GVHD) pathology grading based on IELs infiltration and other pathological characteristics have been established. Intraepithelial B-lymphocytes (B-IELs) exist in salivary gland of primary Sjogren’s syndrome (pSS) patients, which act as a clear indicator of pSS and could be used as an objective alternative to scoring of striated ducts with hyperplasia (10). Furthermore, it is acknowledged that the complex microbial environment presents in the oral cavity, including various carrier molecules and cytokine networks associated with different oral diseases. These factors may play a role in the activation and function of IELs in oral diseases.

In this review, the characteristics of IELs was outlined and novel insights into the potential role of IELs in human oral diseases, including OLP, OSCC, PD, GVHD, and pSS were provided.

## Subsets and development of IELs

**Figure 1:**
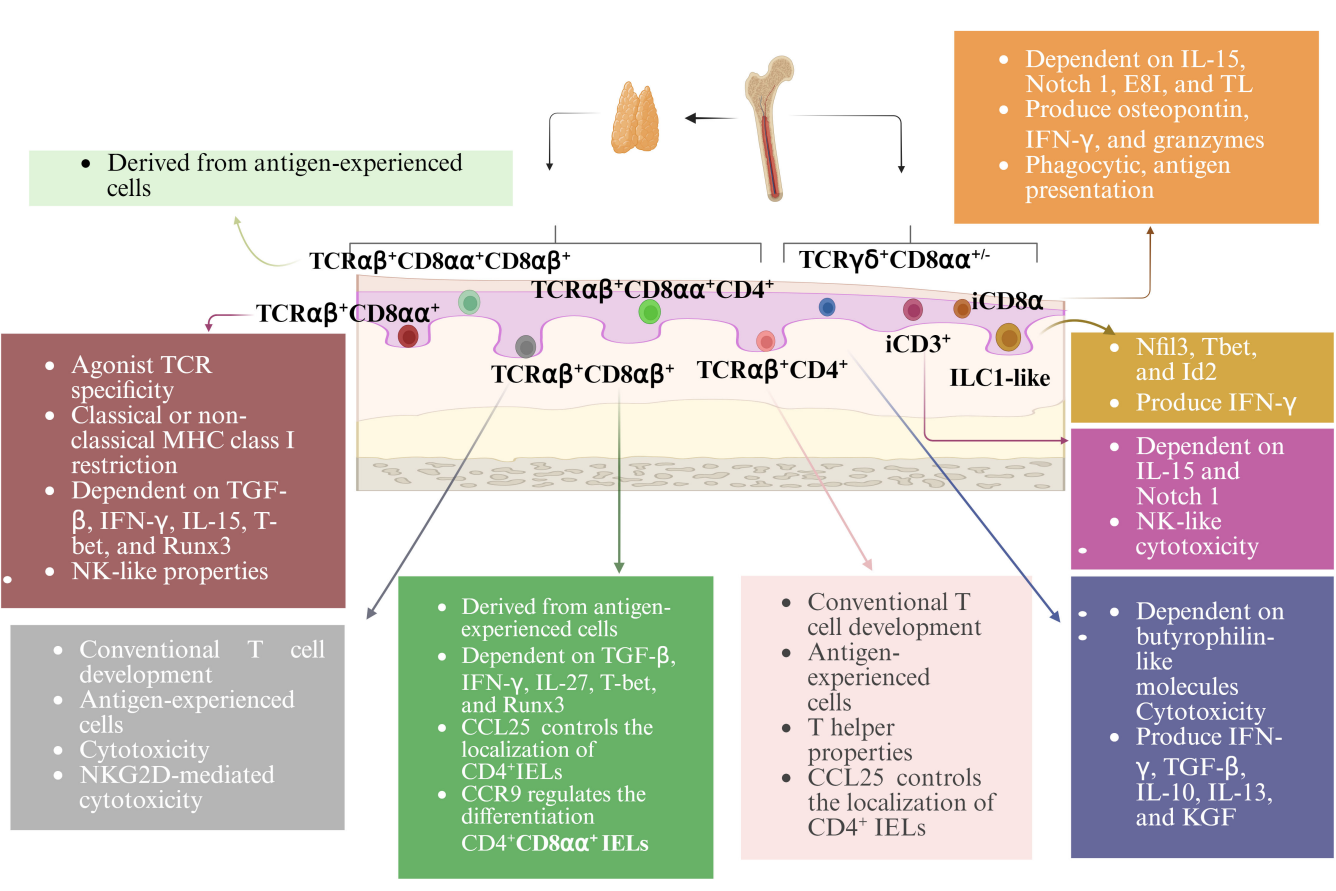
Subsets and development of IELs. With the exception of TCRγδ^+^ IELs, all TCR^+^ IELs develop in the thymus. All TCR^-^ IELs develop extrathymically. Induced TCR^+^ IELs follow a conventional thymic development and the selection pathway, whereas natural CD8αα^+^TCRαβ^+^ IELs undergo agonist selection. IELs require a variety of transcription factors for their development and function. Many TCR^+^ IELs initiate CD8αα expression upon entry into the epithelium. The development, maintenance, and homeostasis of IELs require a variety of factors.

Approximately 90% of all IELs are TCR^+^. These cells can be further divided into induced and natural TCR^+^ IELs (also called conventional or type a and unconventional or type b IELs, respectively (11)). Induced TCR^+^ IELs are derived from conventional Ag-specific T cells that were activated in the periphery and subsequently entered the epithelium. This group of IELs includes CD4^+^ and CD8αβ^+^ subsets. Natural TCR^+^ IELs include TCRαβ^+^ and TCRγδ^+^ subsets, which immediately enter the IELs compartment following their generation. TCR^-^ IELs include subsets resembling innate lymphoid cells (ILCs) that are found outside the intestinal epithelium. Humans contain ILC1-like IELs expressing NKp44 (NCR2/CD336) (12–14). A subset of IELs that express NKp44 and resemble peripheral ILC3 cells have also been identified in humans (15). Another subset of TCR^-^ IELs, present in mice and humans, express intracellular CD3 (iCD3) chains and are called iCD3^+^ TCR^-^ IELs (16). One subset of TCR^-^ IELs, identified in mice and humans, express iCD3 chains together with surface CD8αα and are referred to as innate CD8αα^+^ (iCD8α) cell (16–18) (**Figure 1**).

Induced TCR^+^ IELs are derived from conventional age experienced T cells that enter the intestinal epithelium. Their ontogeny follows the conventional intrathymic development pathway. Natural CD8αα^+^TCRαβ^+^ IELs undergo agonist selection. Being consistent with their lack of Ag-specific receptors, TCR^-^ IELs develop extrathymically (19). The development of murine NKp46^+^ ILC1-like IELs is dependent on the transcription factors Nfil3 and Id2 (12), which requires for the development of all peripheral ILC subsets. The development of these cells also requires T-bet expression (12) (**Figure 1**).

## Activation and maintenance of IELs

**Figure 2:**
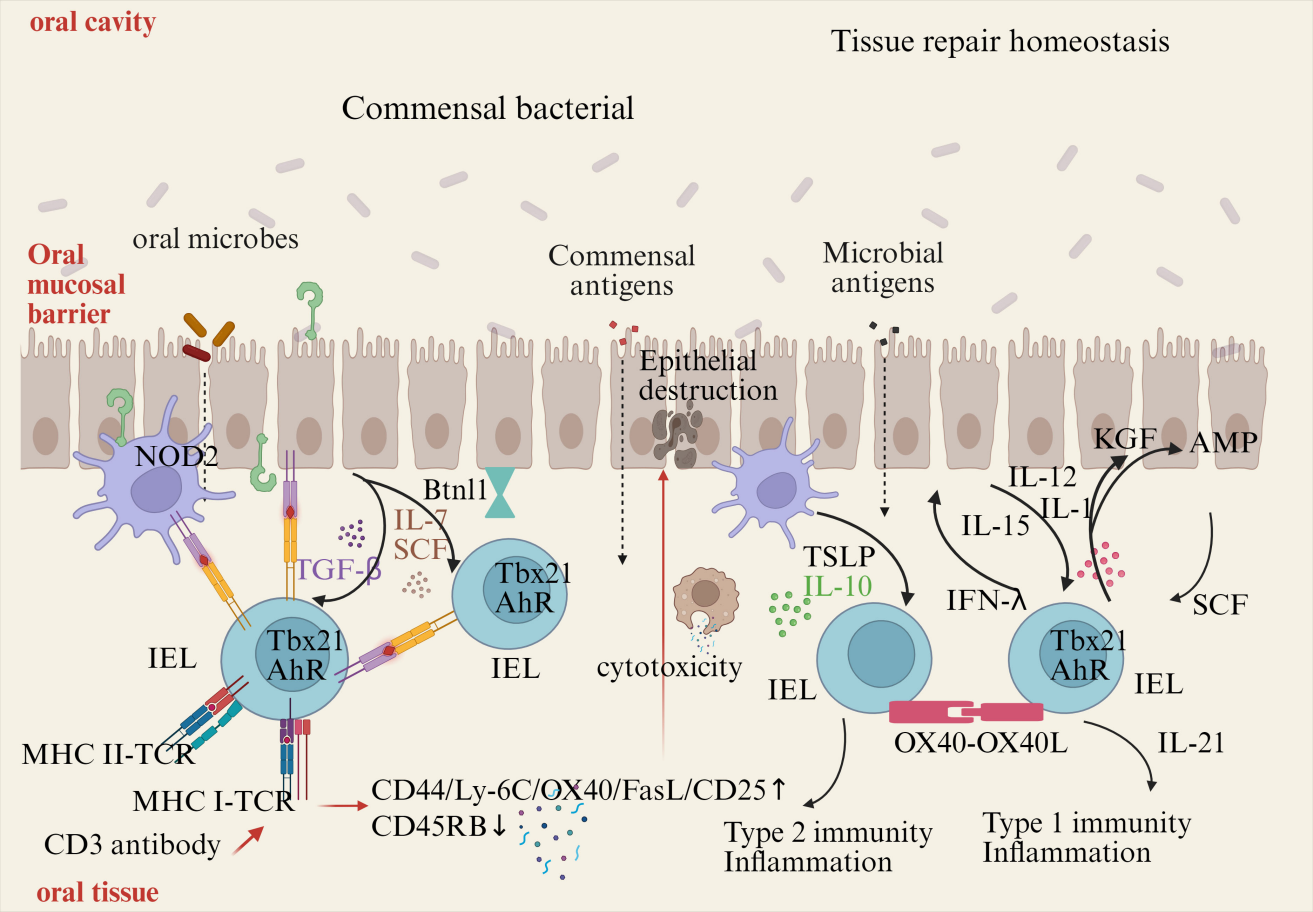
Activation and maintenance of IELs. Commensal bacteria can contribute to IELs maintenance. Signaling via TLR2 and myeloid differentiation primary response gene 88 (MyD88) increases IL-15 production, an important survival factor for IELs. Antigen presenting cells, such as dendritic cells (DCs) or macrophages, also produce IL-15 in a NOD2 dependent manner. IL-15 is bound to the IL-15Rα on the producing cells, and is presented in trans to the IELs, which carries the IL-15Rβ/Cγ chain receptor complex, and signals via the transcription factor Tbx21. IL-7 and stem cell factor (SCF) are additional examples for IEC derived cytokines important for IELs survival, while arylhydrocarbon receptor expression (AhR) and tissue-specific factors, such as butyrophilin-like 1 (Btnl1), play an additional role in maintaining IELs. Infections cause disruption or damage to the epithelial barrier. Dependent on the type of insult, IEC and DCs produce cytokines like thymic stromal lymphopoietin (TSLP), IL-10, IL-12, or SCF, thereby directing the type of immune response. Additional stimulation may be derived from IEL-IEL cross-talk, such as via OX40-XO40L interactions. IELs produce pro-inflammatory cytokines such as interferons (IFNs) and tumor necrosis factor (TNF), and cytotoxic factors such as Fas ligand (FasL) and granzymes, as well as antimicrobial peptides (AMPs) to contain the infection and contribute to wound healing and restoration of homeostasis by secreting growth factors such as KGF. Aberrant IELs activation and potentiation by cytokines might be involved in the development of chronic inflammation.

*In vivo*, the activation IELs can be achieved, at least in part, by TCR ligation. Positive selection of the agonist-driven IELs in the thymus suggests that the mature IELs at the epithelial barrier can subsequently be activated by specific TCR ligands. TCR activation of IELs can be achieved by cell surface receptors, such as non-classical MHC molecules (20, 21). After TCR signaling complexes are stimulated by anti-CD3 antibodies, the balanced IELs show higher expression of CD44, Ly-6C, OX40, FasL, and CD25, and reduced CD45RB protein expression, which indicates the expression of both cytotoxic mediators and cytokine transcripts (22, 23)(**Figure 2**).

Cytokines are essential for IELs activation. IELs rapidly produce effector cytokines and cytotoxic molecules, such as keratinocyte growth factor (KGF), insulin-like growth factor 1 (IGF1), and transforming growth factor (TGF-β) in the stage early activation (24). What’s more, the expression of KGF is limited to γδ^-^τCR^+^ IELs. IELs can express the receptors of TNF, leukemia inhibitory factor, thymic stromal lymphopoietin (TSLP), stem cell factor (SCF; c-Kit ligand), TGF-β, IL-12, IL-15, and IL-1. TGF-β is required for the maintenance of native CD8αα IELs and the expression of CD103. High levels of IL-15 can trigger CD8^+^ T cells to become cytotoxic independently of the TCR (25, 26). The stimulation of IL-15 on IELs results in increased production of IFN-γ and TNF, granzyme-dependent cytotoxicity, and enhanced expression of NK receptors (27).

IELs constitutively express transcripts of genes, which are associated with activated cytotoxic T cells, such as granzyme A, granzyme B, serine, Fas ligand (FasL), and chemokine C-C motif ligand (CCL). IELs express cytotoxic T lymphocyte-associated protein 4, Ly49E-G, NK cell inhibitory receptor Ig superfamily-related gp49B, and programmed cell death 1 (PD-1) (28). Unlike conventional CD8^+^ T cells, IELs express high levels of Tnfsf6 transcripts during steady state (29), but do not express the protein encoding FasL on their surface until additional activation occurs (29, 30).

Maintaining IELs requires the involvement of multiple cytokines and signaling pathways. The ligand-activated transcription factor and the aryl hydrocarbon receptor (AHR) are essential for the maintaining of IELs (31, 32). Maintenance and activation of IELs also critically depend on the interaction between epithelial cells and microbes. The myeloid differentiation primary response gene 88 (MyD88) can activate NF-κB, which is essential for IELs maintenance (33). IL-15 produces signals by expressing the type 1 transcription factor T-box in T cells (T-bet) to sustain IEL precursors (34). Protein containing a nucleotide-binding oligomerization domain (NOD) 2 is also crucial for maintaining IELs (35) (**Figure 2**).

## IELs and human oral diseases

The oral mucosa consists of stratified squamous epithelium, lamina propria (LP) and MALT, mucosal-associated lymphoid tissue (MALTs). Dendritic cell (DCs), Langerhans cells (LCs) and, intraepithelial lymphocyte (IELs) reside in the epithelial layer. Numerous microorganisms and antigens and stimuli from exogenous substances (36) such as food undergo sophisticated regulation by immune responses in the oral mucosa. T cells especially IELs are crucial for this defense (37). CD8αα^+^ IELs in the oral-pharyngeal mucosa have not been identified and characterized until recently year. And TGF-β regulates the development/generation of oral CD8αα^+^ IELs (38).

## IELs in oral lichen planus

OLP is a chronic inflammatory disease that occurs in the oral mucosa and is labelled as oral potentially malignant disorder (OPMD) by the World Health Organization (WHO). Although the etiology is unknown, T-cell-mediated immune response is pivotal in the pathogenesis of OLP. T cells infiltrate the lamina propria, secrete cytokines to construct an inflammatory environment and mediate keratinocyte apoptosis, participating in the development of OLP.

OLP is characterized by subepithelial lymphocyte infiltration and elevated IELs (4). Most subepithelial and intraepithelial lymphocytes are CD8^+^ T cells (4). The infiltrates in the lamina propria of OLP lesions are mostly CD4^+^ Th cells, while T cells near the basement membrane region are dominated by cytotoxic CD8^+^ T cells (4). There is approximately a 1:2 ratio of CD4^+^ IELs/CD8^+^ IELs in OLP (39). Our previous study identified a significant infiltration of elongated CD8α^+^ IELs in the basal cell layer of OLP (40). Enomoto A et al. proposed that CD8^+^ IELs could serve as a predictive biomarker for OLP remission. They found that CD8^+^ IELs were associated with the remission rate in a subgroup that exhibited higher T-bet/FOXP3 subset balance (indicating inducible cytotoxic immunity) and determined a predicted cut-off value for CD8^+^ IELs (16 cells/high-power fields) (41).

Gene expression analysis of OLP oral mucosa samples disclose increased transcript expression of killer cell lectin-like receptor subfamily G member 1 (KLRG1), CD8A, and granzyme K (GZMK) (42). CD8 T cells and KLRG1^+^ T cells localized within the intraepithelial regions, both at the basal layers and more superficially and often adjacent to keratinocytes (42). Cytotoxic CD8^+^ IELs cluster in areas of basement membrane disruption (43) and adjacent to degenerating keratinocytes (44), which indicates that CD8^+^ IELs may be engaged in epithelial cell destruction by typical cytotoxic mechanisms of CD8 T cells in OLP, such as release of granzymes and direct killing of target cells.

CD8A encodes the CD8α chain that is a cell surface glycoprotein expressed on cytotoxic T cells, and it plays a pivotal role in antigen recognition and the immune response against infected or abnormal cells (45). Research indicates that CD8αα is a crucial modulator of the IEL’s dynamic migration between the inner epithelium and the lamina propria. While CD8ααCD4 IELs were in the lamina propria, adoptively transferred CD8αCD4 IELs were found within the recipient mice’s epithelium (46).

CD8 IELs are often tissue-resident memory T (Trm) cells in OLP lesions. Compared to nonerosive oral lichen planus (NEOLP), CD8^+^ Trm cells was increased in erosive oral lichen planus (EOLP), which were adjacent to the epithelium and its products may induce epithelial erosion. CD8^+^ Trm cells in particular exhibited higher expression levels of GZMA, GZMK, TNF, PRF1, and other genes associated with inflammatory factors, when compared to other subgroups. It may have contributed to the worsening of the clinical manifestations of OLP. CD8^+^ Trm marker gene CD69, GNLY which can play a cytotoxic role, and multiple pro-inflammatory factor-related genes, such as GZMB, IFNG, TNF, and PRF1, were significantly increased in the CD8^+^ Trm subgroup in EOLP (47).

The expression of TNF, IL17A/IL17RA, IFNGR1, etc. was higher in EOLP than in normal oral mucosa. And the signals of IFNGR1 and IL17RA were significantly enhanced in EOLP compared with NEOLP. CD8^+^ Trm cells in EOLP produced significantly higher levels of TNF-α, IFN-γ, and IL-17 than those in NEOLP, with the increase in IFN-γ being statistically significant. Therefore, CD8^+^ Trm cells may affect the clinical manifestations of OLP through the secretion of IFN-γ (47).

Biologic therapies targeting cytokines such as anti-TNF-α, anti-IL17, and anti-IL12/23 have been employed with variable outcomes. TNF-α inhibitors (etanercept, infliximab, and adalimumab) have shown promise. Additionally, therapies such as Alefacept and agents targeting IL-17 and related pathways (e.g., ustekinumab, guselkumab, secukinumab, and tildrakizumab) have demonstrated efficacy, particularly in reducing the Th1 and Th17/Tc17 cellular mucosal infiltrate, suggesting a key role for IL-17-producing T cells in OLP pathogenesis (48).

The integrin αE (CD103) β7 (αEβ7) is expressed by IELs, dendritic cells and regulatory T cells, and mediates cell migration and homing (49). The percentage of CD103 γδT cells was upregulated in OLP γδ IELs are the main group of IELs with highly motility (50). The interaction between the IELs and the pathogen is critical for γδ IELs surveillance and direct host defense (51). TCRγδ^+^ IELs recognize autoantigen molecules expressed by epithelial cells to activate the NKG2D receptor pathway and play a dynamic defense role in the epithelium (52). IL-15 secreted by epithelial cells can induce NKG2D activation and upregulation on the surface of IELs, thus preventing the activation of the inhibitory receptor NKG2A and then activating the ability of IELs to destroy epithelial cells (17, 53). Research has found the co-localization of IELs with IL-7 secreted by epithelial cells (11), which suggests that IL-7 that is secreted by epithelial cells may mediate the close interaction between IELs and epithelial cells in OLP.

MAIT cells express transcription factors retinoic acid receptor-related orphan receptor gamma-t (RORγt) and T-bet, regulating the secretion of IL-17, IFN-γ. etc (54–56). Additionally, MAIT cells can secrete Th2-type cytokines like IL-13 under chronic inflammation stimulation (57). This cytokine secretion potentially enables MAIT cells to modulate Th1, Th2, and Th17 cells, suggesting a potential immunomodulatory capacity exerted by MAIT cells. In OLP patients, TNF and IFN-γ upregulate endothelial adhesion molecules like CD31, CD106, CD54, and CD62E in blood vessels and stimulate the production of the chemokine CCL5 by keratinocytes (58–60). Circulating T cells are recruited to OLP lesions through these adhesion molecules and chemokines, and the release of TNF and IFN-γ by activated MAIT cells may participate in these processes, thereby promoting T cell recruitment.

Current studies on the interaction between MAIT and CD8^+^ T cells primarily focus on vaccine research. Provine et al. highlighted the ability of MAIT cells to sense immune activation signals triggered by viral vectors and integrate them to augment CD8^+^ T-cell responses, with locally produced chemokine CXCL20 likely playing a significant role in this process (61). Additionally, IFN-γ promotes CD8^+^ T cell activation and maintains MHC II expression, with MAIT cells potentially involved in this process, thereby modulating the OLP inflammatory response (62).

## IELs in oral squamous cell carcinoma

Oral squamous cell carcinoma is the most prevalent type of oral cancer, with a 5-year survival rate of approximately 50%. The significantly high rates of local recurrence and cervical lymph node metastasis complicate surgical removal of OSCC, leading to poor prognoses and posing significant threats to human health and well-being. The pathogenesis of OSCC has not been fully elucidated, which is a consequence of complicated multiple-factors synergetic effects and associated with the changes of oncogenes and tumor suppressor genes and a series of tumor immunological responses (63–65).

IELs infiltration and expression of tumor related factors were observed in OSCC. In lip carcinogenesis, there was an increase in peritumoral and intratumoral CD3^+^, CD8^+^, CD20^+^ and CD68^+^ cells. In the intraepithelial region, CD8^+^ cells are correlated with CD20^+^ and CD68^+^ cells (66). In tongue cancers, tumor nest-infiltrating CD8^+^ IELs frequently expressed PD-1, an inhibitory receptor, in sharp contrast to those in the stroma or in the lichen planus. Conversely, CD8^+^ IELs only infrequently expressed NKG2D, an activating receptor, in contrast to those in the stroma or in the lichen planus. No CD8^+^ IELs expressed Ki-67, a proliferation associated marker, whereas those in the stroma frequently expressed it (67). CD8^+^ IELs in tongue cancer tumor nests was phenotypically inactivated, which indicated the first immune escape in OSCC tumor nests (67).

FOXP3^+^ IELs were significantly increased in OSCC patients (68). Tumor-infiltrating FOXP31^+^ IELs were significantly more frequent in oropharynx cancer and OSCC and patients without lymph node metastasis (68). Additionally, high infiltration of regulatory FOXP31^+^ IELs and relatively high levels of BDCA21 and FOXP31 cells in stromal (peripheral) regions of the tumors were found in head and neck squamous cell carcinoma (68).

A higher number of CD8^+^ T cells was significantly associated with poorer outcome. In the tumor-bearing part of involved lymph node tissue, more CD8^+^ T cells were observed than in primary cancer. CD8^+^CD103^+^ Trm cell infiltration in T2 tumors was higher than in T1 or T4 tumors (69). However, patients with a higher density of CD8^+^ T cells in their cancer survive longer than patients with lower numbers. Patients with Trm cell-high cancers had better overall survival than patients with Trm cell-low tumors. In addition, Trm cell infiltration was absent in metastatic disease or at recurrence, serves as a marker of better survival. The checkpoint molecule TIM3, was expressed significantly higher on Trm and non-Trm cells in the lymph node compared with primary tumors, which was also seen in recurrences. The role of TIM3, as a therapeutic target remains to be defined (69).

The early progression of oral precancerous lesions to cancer was enhanced in IL-23 receptor-deficient mice, which suggested the importance of IL-23. Exogenous IL-23 can promote the activation of CD8^+^ IELs with high expression of IL-23R (70). IL-23 is known to modulate the homeostasis of neutrophil infiltration into tissues by inducing expression of IL-17 and G-CSF18, which are linked to tumor growth (71). IL-23 can induce IL-17 production by tumor-resident immune cells, including CD4^+^ Th17 cells, natural killer T (NKT) cells, γδ T cells and CD8^+^ cytotoxic T lymphocytes (CTLs). IL-23 also opposes the action of IFN-γ and the subsequent production of cytotoxic mediators such as perforin, granzymes, and Fas ligand (FasL), and can also inhibit IFNγ-mediated MHC-I upregulation (72).

The duction of IFN-γ by T-cell induction by IL-23 antagonized the local inflammatory response as well as IELs infiltration in the tumor immune microenvironment (TME) (73). Low-density CD4FOXP3 IELs infiltration was observed within the OSCC invasion front and tumor center, which suggested a poor prognosis. The rate of local failure in older cancer patients improved with increasing levels of CD3^+^ IELs and CD8^+^ IELs (74). In low-risk oropharyngeal and hypopharyngeal SCC, high infiltration of CD8^+^ IELs may improve disease-free survival (75). The cytotoxic activity and tumor infiltrating ability of CD8^+^ T cells might be largely inhibited owing to a local protective tumor microenvironment induced or fostered by IL-23.

## IELs in periodontal disease

Periodontal disease is a chronic inflammatory disease characterized by an inflammatory environment, mainly affecting the gingiva, bone, and ligament (76). Periodontal disease includes gingivitis and periodontitis.

Junctional epithelium (JE), the first line of periodontal defense against bacterial infection, constitutively expresses ICAM-1, cytokines and chemokines, together to maintain the physiological homeostasis of JE (77). IELs are localized to the middle layer of JE, in which the number of TCR^+^ lymphocytes is higher than that in systemic immune organs, such as spleen and lymph nodes (78). IELs in JE express TCR and CD3 in conventional and germ-free mouse (79). TCR-positive T cells constitute the main population of IELs (80, 81).

The gingiva contains a significant population of Vγ6^+^ γδ T cells (82, 83). γδ IELs are the first line of defense against luminal microorganisms and they are adjacent to dental biofilm, which implies their possible role in the host-microbiota interactions in the gingiva (82). The microbiome is both necessary and sufficient for the observed increase in γδ IELs (82, 84, 85). Furthermore, adult mice treated with antibiotics had a substantial decrease in the frequency of γδ IELs in their gingiva (82), which indicated that the microbiota may have impacts on the development and maintenance of γδ IELs (85).

γδ IELs are involved in regulating the oral microbiota (85) and can induce a large amount of IL-17, which mediates the development and progression of periodontitis (8). IL-17 plays a predominantly protective role in periodontal diseases (86). Vγ6^+^γδ T cell can produce large amounts of IL-17A to accelerate bone formation at the fracture site by stimulating the proliferation of mesenchymal progenitors and the differentiation of osteoblasts (87). However, some studies have shown that the presence of γδ T cells and IL-17 in periodontal tissue is positively associated with the severity of periodontitis, which may be related to the ability of IL-17 to recruit inflammatory cells (88, 89). γδ T cells can inhibit periodontal bone loss and promote gingival repair by producing restorative cytokines, such as amphiregulin (a member of the epidermal growth factor family) (90, 91).

## IELs in graft-versus-host disease

GVHD is a common and significant complication of allogeneic hematopoietic cell transplantation (allo-HCT). Both apoptotic bodies and IELs were increased in the gallbladder of patients with HCT (9). Oral chronic GVHD (cGVHD) occurs after approximately 70% of HCT, including lichenoid mucosal responses, restricted mouth opening, and salivary gland dysfunction. According to literature reported, patients with refractory Hodgkin’s lymphoma developed tongue GVHD after receiving allo-HCT. Tongue biopsy showed changes in moss and keratinized tissue, accompanied by epithelial T cell infiltration, which was consistent with cGVHD (92).

A large cohort analysis on histopathological grading of oral mucosal cGVHD indicated that a points-based grading tool (0 to 19, grade 0 to IV, 0→IV: mild→severe) was established. The evaluation indicators of this tool include IELs and band-like inflammatory infiltrate, atrophic epithelium with basal cell liquefaction degeneration, including apoptosis, as well as separation of epithelium and pseudo-rete ridges. From grade 0 to IV, IELs infiltration ranged from *no/occasional* to *widespread (* 93). The grade II-IV biopsy specimens demonstrated a histopathological diagnosis of active mucosal lichenoid-like cGVHD, which highlighted the importance of correlating clinical presentation with the dynamic histopathological processes for improved patient stratification. Most importantly, this tool could be used for assessing treatments, pathological processes, and immune cellular content to provide further insights into this debilitating disease (93). Recently, the histological NIH cGVHD grading for defining features of salivary gland cGVHD (sg-cGVHD) with awarded points was designed (**Table 1**) (94). Peri-ductal and acinar lymphocytic infiltration is an important consideration in the NIH cGVHD grading form.

**Table 1:** (94) Grading of minor salivary gland immuno-histopathology post-allogenic hematopoietic cell transplantation.

Type of inflammation				
Feature	a) None	b) Lymphocytic	c) Plasmoctic	d) Chronic mixed
n (%)	12 (11.7)	8 (7.7)	12 (11.7)	71 (68.9)
**Ducts**				
**Features and points**	**None = 0**	**Mild = 1**	**Marked = 2**	
**1. Periductal infiltrate n (%)**	Sporadic 17 (16.5)	Focal 53 (51.5)	Widespread 33 (32.0)	
**2. Periductal exocytosis n (%)**	None 58 (56.3)	Focal 31 (3s0.1)	Widespread 14 (13.6)	
3. Ductal damage* n (%)	None 57 (55.3)	Focal 31 (30.])	Widespread 15 (14.6)	
**4. Periductal fibroplasia n (%)**	Discrete 29 (28.2)	Some 68 (66.0)	Intense 6 (5.8)	
**Acini**				
**Features and points**	**None = 0**	**Mild = 1**	**Marked = 2**	
**5. Peri-acinar infiltrate n (%)**	Sporadic 18 (17.5)	Focal 57 (55.3)	Widespread 28 (27.2)	
**6. Acinar exocytosis n (%)**	None 73 (70.9)	Focal 28 (27.2)	Widespread 2 (1.9)	
7. Acinar destruction ** n (%)	None 28 (27.2)	Focal 45 (43.7)	Widespread 30 (29.1)	
**8. Interstitial fibrosis n (%)**	None 22 (21.4)	Some 60 (58.2)	Intense 21 (20.4)	
**Total points**: 16 (Grade 0 - 0-2; Grade I - 3-4; Grade II - 5-7; Grade III - 8--11; Grade IV - 12-16)				
GO; n = 13 (12.6%)	GI; n = 28 (27.2%)	Gll; n = 24 (23.3%)	GIII; n = 21 (20.4%)	GIV; n = 17 (16.5%)
Median points: 1	Median points: 2	Median points: 5	Median points: 10	Median points: 13

*Damage represents vacuoler changes and apoptosis.

**Destruction represents atrophy, ductal metaplasia and apoptosis.

Assessment of MSG 103 biopsies that included 100 biopsies from HCT patients and three healthy control biopsies.

Histological MSG evaluation was based on the NIH cGVHD Pathology resource document (“NIH cGVHD grading”) with associated NIH specific criteria for sg-cGVHD. The NIH cGVHD grading form was designed to cover degree of peri-ductal and acinar infiltration, including exocytosis, ductal damage, and acinar degeneration, as well as peri-ductal and interstitial fibrosis (in bold font). Each feature was assessed as mild or marked with a final pathological scoring range, and points for the features were allocated (0–16 points).

High expression of CD4, CD8, and FOXP3 in GVHD confirmed that oral cGVHD was primarily driven by T cells and involved by macrophages (95). The presence of a CD4/CD8 double-positive T cell population in adult allo-HCT recipients was predictive of grade II GVHD (96). Research proved that Notch signaling promoted T cell pathogenesis and GVHD after allo-HCT, in which δ-like Notch ligand DLL4 played a dominant role. IELs expressing CD3 in human and mouse can differentiate along the transcription factor Id2-independent pathway under NOTCH1 signaling (16), which is involved in the occurrence and development of GVHD.

## IELs in primary Sjogren’s syndrome

pSS is a chronic inflammatory autoimmune disease that primarily affects the function of exocrine glands such as salivary and lacrimal glands. In tissues of pSS, lymphocytes infiltrate into salivary and lacrimal glands to produce autoantibodies (97).

Intraepithelial T-lymphocytes (T-IELs) scattered throughout the striated ductal epithelium of salivary glands of pSS. Intraepithelial B-lymphocytes (B-IELs) were found in almost all striated ducts with hyperplasia in lymphoepithelial lesions (LELs). B-IELs and B-IELs/T-IELs ratios increased significantly with higher severity of LELs, which was even more pronounced in the parotid than in the labial gland (98). The presence of B-IELs in salivary gland biopsies patients is a clear indicator of pSS and can be used as an objective alternative to LEL scoring (10).

Fc receptor-like protein 4 (FcRL4) is normally expressed on a small subset of mucosa-associated B-cells, as well as on MALT lymphoma B-cells. pSS patients have an increased risk of developing MALT lymphomas, preferentially in the parotid glands. FcRL4 mRNA expression level in parotid MALT lymphoma is increased compared to parotid gland tissue of pSS patients without lymphoma. However, numbers of FcRL4^+^ B-cells in labial gland biopsies taken at the time of pSS diagnosis, are not predictive for later development of MALT lymphoma (99). Pathway analysis showed upregulation of B cell activation, cell cycle and metabolic pathways. FcRL4^+^ B cells are expected to be an important treatment target in pSS (100).

In pSS, ductal cells produce a wide variety of cytokines (101), which can contribute to the activation of the FcRL4^+^ B-IELs, and, particularly, FcRL4^+^ B-IELs express the highest levels of FcRL4.

FcRL4^+^ B-IELs have strong expression of CD20 that makes them highly susceptible targets for rituximab therapy (102, 103). Treatment with rituximab did not only reduce the total number of B cells, but also FcRL4^+^ B-IELs, which resulted in a significant decrease in the number of LELs and normalization of the epithelial lining (99, 104). Apparently, when FcRL4^+^ B-IELs are depleted from the epithelium, stimulation of ductal cells by FcRL4^+^ B-IELs is no longer present, which enables the restoration of epithelium. Of note, blocking the CD28 mediated co-stimulation with abatacept did not affect the presence of FcRL4^+^ B-IELs and the numbers and severity of LELs concomitantly (105).

Therefore, it has been proposed that identification of B-lymphocyte–containing ducts should be added to the diagnostic histopathological work-up of patients suspected of pSS (10).

## Conclusion and the future development direction

**Figure 3:**
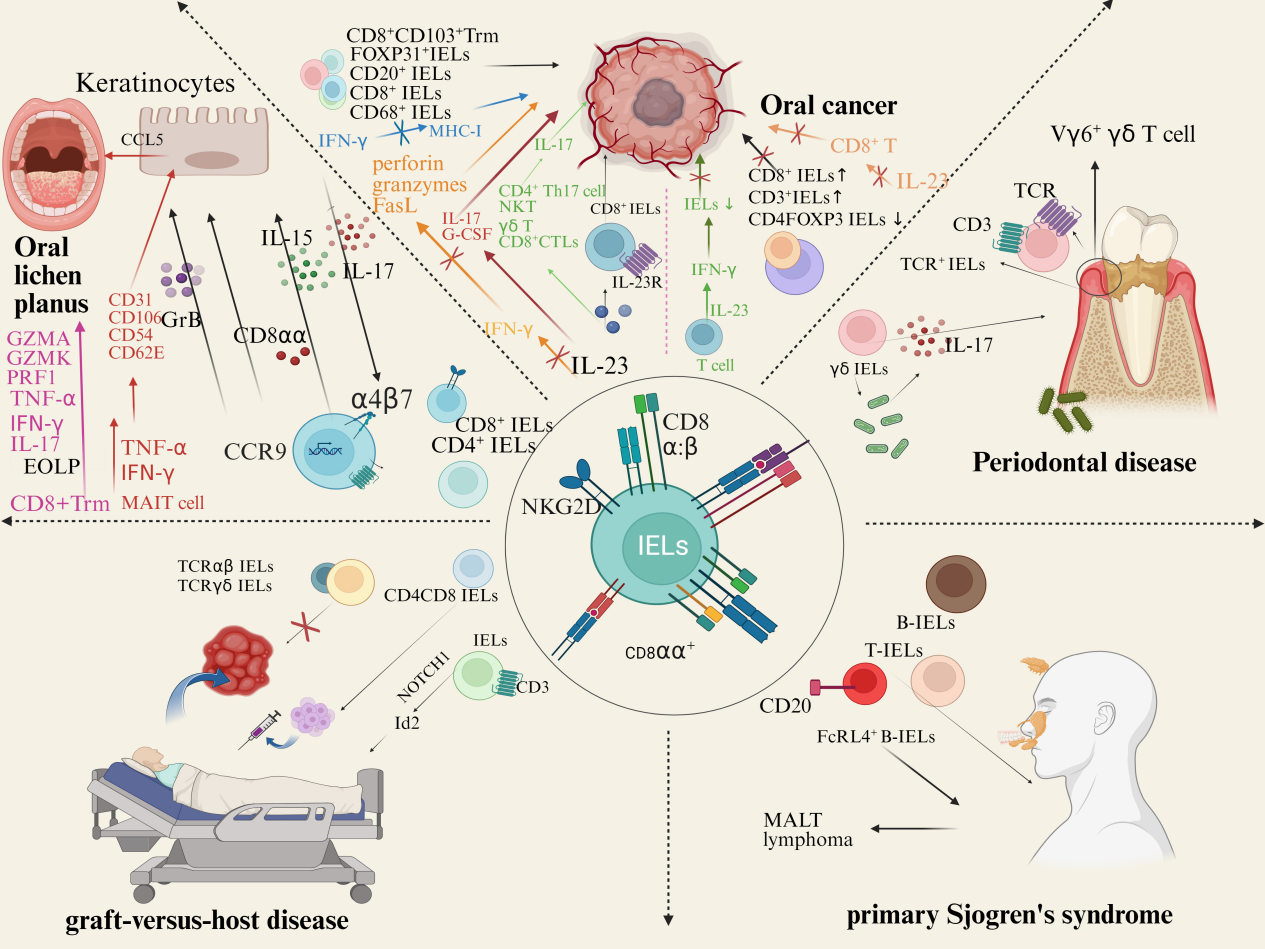
IELs and human oral diseases. There is approximately a 1:2 ratio of CD4^+^IELs/CD8^+^IELs in OLP. CD8αα and Integrin αE (CD103) β7 (αEβ7) mediates cell migration and homing. TCRγδ^+^ IELs recognize autoantigen molecules expressed by damaged keratinocytes, activate the NKG2D receptor pathway. IL-15 secreted by epithelial cells can induce NKG2D activation and upregulation on the surface of IELs, preventing the activation of the inhibitory receptor NKG2A. Co-localization of IELs with IL-7 secreted by epithelial cells. In lip carcinogenesis, peritumoral and intratumoral CD3^+^, CD8^+^, CD20^+^ and CD68^+^ cells increase. In tongue cancers, CD8^+^ IELs frequently expressed PD-1. IL-23 promote the activation of CD8^+^ IELs. IELs in junctional epithelium express TCR and CD3. Most gingival γδ IELs are adjacent to dental biofilm. γδ IELs induce a large amount of IL-17, which mediates the development and progression of PD. IELs were increased in the gallbladder of patients with HCT. High expression of CD4, CD8, and FOXP3 in GVHD confirmed that oral cGVHD is primarily driven by T cells. IELs expressing CD3 in human and mouse can differentiate along the transcription factor Id 2-independent pathway under NOTCH1 signaling. TCRαβ/CD19 cell depletion is often used in HCT. γδ T cells may undergo CXCR4 signaling to recruit alloreactive CD4 T cells to target tissues. B-IELs were found in almost all striated ducts with hyperplasia in lymphoepithelial lesions (LELs). FcRL4^+^ B-IELs are present in the salivary glands of pSS patients. B-IELs/T-IELs ratios increased significantly with higher severity of LELs.

The increasing evidence provides new insights into the role of IELs in the pathogenesis of OLP, OSCC, PD, GVHD, and pSS (**Figure 3**). Notably, IELs are increased in OLP lesions, thus killing epithelial cells directly or indirectly through cytotoxicity and destroying the basement membrane. IELs are a double-edged sword in OSCC. The infiltration of IELs around and within the tumor of OSCC has the potential to promote the tumor growth, and immune molecules related to regulating its cytotoxicity may regulate the prognosis of OSCC. However, in low-risk OSCC, survival could be improved due to IELs. IELs in junctional epithelium are involved in regulating the host-microbiota interactions to mediate the development of periodontal disease. Additionally, IELs in glands are increased in patients with pSS and are involved in the destruction of the gland and ductal tissue. However, the exact role played by IELs in the occurrence and development of oral diseases is still largely unknown. Future studies should not only investigate the biological functions and precise molecular mechanisms of IELs in oral diseases, but also address the clinical applications of IELs, aiming to facilitate the clinical translation.
